# Connections between Cognitive Impairment and Atrial Fibrillation in Patients with Diabetes Mellitus Type 2

**DOI:** 10.3390/biomedicines12030672

**Published:** 2024-03-17

**Authors:** Marius Militaru, Daniel Florin Lighezan, Cristina Tudoran, Anda Gabriela Militaru

**Affiliations:** 1 Department VIII, Neuroscience, Discipline of Neurology II, University of Medicine and Pharmacy “Victor Babes” Timisoara, E. Murgu Square, Nr. 2, 300041 Timisoara, Romania; marius.militaru@umft.ro; 2 Municipal Emergency Hospital Timisoara, Gheorghe Dima Street Nr. 5, 300254 Timisoara, Romania; dlighezan@umft.ro (D.F.L.); militaru.anda@umft.ro (A.G.M.); 3 Center of Advanced Research in Cardiology and Hemostasology, University of Medicine and Pharmacy “Victor Babes” Timisoara, E. Murgu Square, Nr. 2, 300041 Timisoara, Romania; 4 Department V, Internal Medicine I, Discipline of Medical Semiology I, University of Medicine and Pharmacy “Victor Babes” Timisoara, E. Murgu Square, Nr. 2, 300041 Timisoara, Romania; 5 Department VII, Internal Medicine II, Discipline of Cardiology, University of Medicine and Pharmacy “Victor Babes” Timisoara, E. Murgu Square, Nr. 2, 300041 Timisoara, Romania; 6 Center of Molecular Research in Nephrology and Vascular Disease, University of Medicine and Pharmacy “Victor Babes” Timisoara, E. Murgu Square, Nr. 2, 300041 Timisoara, Romania; 7 County Emergency Hospital “Pius Brinzeu”, L. Rebreanu, Nr. 156, 300723 Timisoara, Romania

**Keywords:** diabetes mellitus, atrial fibrillation, cognitive decline, dementia, subclinical atherosclerosis, memory tests

## Abstract

(1) Background: Cognitive decline (CD), considered a precursory state of dementia, is frequently encountered in patients with diabetes mellitus type 2 (DM-2) and might even have a higher prevalence in those with associated atrial fibrillation (AF). In this study, we aimed to research if the association of DM-2 and AF favors a precocious onset of CD. (2) Methods: This study was conducted on 160 patients, featuring 50 with DM-2, 54 with DM-2 and AF, and 56 subjects without DM-2 and AF, all evaluated clinically and with five neuropsychiatric scales. (3) Results: The Mini-Mental-State-Examination (MMSE), Montreal Cognitive Assessment (MoCA), Activities of Daily Living Score (ADL), Instrumental Activities of Daily Living Score (IADL), and Geriatric Depression Scale (GDS-15) were significantly altered in patients with DM-2 and AF in comparison to patients without these diseases. The logistic regression model indicated that, in patients with DM-2 and AF, an increase of one year in age is associated with a 7.3% augmentation of the risk of a precocious onset of CD (MMSE < 27). (4) Conclusions: CD is more frequent in patients with DM-2, especially when associated with AF, versus those without DM-2 and AF. Our findings suggest that an older age and associated dyslipidemia represent risk factors for CD in patients with DM-2.

## 1. Introduction

Along with the progressive increase in the populations’ life expectancy, worldwide, healthcare systems have to cope with the burden represented by multiple age-related degenerative disorders, among which cognitive decline (CD) represents one of the most invalidating conditions [1]. This is defined as a decrease in performance in one or more cognitive domains, such as integrated attention, decision-making, learning and memory, communication, perception, motor, or social cognition [2]. The CD includes a broad spectrum according to its impact on a patient’s independence, ranging from mild (when the subject is still capable of caring for him/herself) to severe CD (when this ability is partially lost) [3]. CD represents a precursory state for dementia, which is considered a global challenge, as it alters the patient’s functional status and leads to a progressive loss of independence, with the patient becoming unable to take care of him/herself and having to rely on additional support offered by their family or local social assistance centers [4]. CD remains frequently undetected, especially in elderly patients with associated comorbidities. As individuals with CD have a 10% annual likeliness to develop dementia, it is of utmost importance to detect alterations in cognitive function as soon as possible.

The mechanisms that are responsible for the development of CD are multiple and not entirely understood, but the role of atherosclerosis of the cerebral arteries is generally accepted. Diabetes mellitus type 2 (DM-2) is also considered to favor the early onset of systemic atherosclerosis [4,5]. DM-2 is a common pathology in our society, being called the “global pandemic of the 21st century” by Standl as, in 2017, it reached a global incidence of 8.8%, and its prevalence rises every year [6]. DM-2 affects predominantly adults, between 40 and 59 years, from low- and middle-income countries, possibly due to a higher prevalence of obesity and insulin resistance (IR) in these regions [7]. DM-2 is associated with a 1.5-fold increased risk of developing CD and dementia, and over 45% of diabetic patients show signs of mild CD [8]. DM-2 may favor the early onset of CD through several mechanisms: (1) subclinical and clinical atherosclerosis; (2) neurodegenerative effects of hypo- and hyperglycemia; and (3) IR increases the generation of beta-amyloids in the brain, also determining cerebral neurodegenerative effects by itself. IR is involved in the development of both DM-2 and atherosclerosis, playing a pivotal role in the progression of these pathologies [7,8,9,10,11].

Atrial fibrillation (AF) is another chronic cardiovascular disease (CVD) associated with more advanced age. It is the most frequent arrhythmia, with a prevalence of 1–3% in the general population and even higher (11%) in elderly patients [12,13]. It is associated with an up to 2.4-fold increase in the risk of developing CD or even dementia [14]. Sometimes, AF is fortuitously diagnosed in patients with silent brain infarcts or those suffering an unexpected stroke, but in recent years, CD has also been encountered in patients with AF even without evidence of a stroke [15]. AF may favor the earlier onset of CD mainly by subclinical atherosclerosis in the cerebral small arteries, but cognitive impairment can also occur through brain hypoperfusion, a proinflammatory state, and microhemorrhages in patients treated with chronic anticoagulant therapy, thus leading to cerebral atrophy [3,16,17,18].

In recent decades, due to the increasing prevalence of both DM-2 and AF worldwide, these pathologies are frequently diagnosed in the same patient [19,20]. Although it is known that DM-2 and AF represent individual risk factors for CD, there a few studies have analyzed their combined effect. There is an urgent need for a better understanding of the factors responsible for the development of cognitive impairment among patients with DM-2 and AF, as well as of the potential underlying pathophysiological mechanisms, in order to develop superior strategies for the management of DM-2 and AF, thus preventing the occurrence of CD and its progression to dementia among these patients.

Our study aims to determine the impact of different risk profiles on the precocious onset of CD, and on its severity, focusing on the association of DM-2 with AF.

## 2. Materials and Methods

### 2.1. Study Population

From all patients admitted between 5 January 2022 to 31 December 2023 in the internal medicine or the neurology department of the Municipal Emergency Hospital Timisoara for CVD or neurologic pathology, 104 subjects with DM-2 who showed signs of CD but had not been yet diagnosed with CD or dementia (and were not treated for these pathologies) met our inclusion criteria. We selected an age-matched group of 56 subjects with CVD but without DM-2 and/or AF. All patients received optimal medical therapy for their stable, chronic conditions, including statins, and those with DM-2 were treated with oral glucose-lowering medication. According to the presence of AF and/or DM-2, we divided our patients into 3 groups: Group A—50 patients with DM-2, Group B—54 subjects with DM-2 and AF, and Group C—56 age-matched subjects, without AF and DM-2, but with chronic CVD or with a very high cardiovascular risk profile (CVRP).

Inclusion criteria were: (1) age over 45 years; (2) signs of cognitive impairment without a previous diagnosis of CD or dementia; (3) history of CVD, and/or DM-2; (4) patients with very high CVRP.

Exclusion criteria: (1) unable or unwilling to sign the informed consent form; (2) uncontrolled DM-2 requiring insulin therapy; (3) acute decompensated CVD: acute myocardial infarction, acute heart failure, etc.; (4) other acute or decompensated pathologies; (5) uncontrolled systemic hypertension (SH); (6) patients with low compliance to the recommended lifestyle measures and medication; (7) chronic unhealthy habits, such as alcohol and drug abuse; (8) significant previously diagnosed mental illnesses; (9) without CD.

All patients underwent a detailed clinical examination in tandem with cardiologic and neurologic evaluations. The clinical assessment consisted of medical history, clinical examination, registration of systolic blood pressure (SBP), diastolic blood pressure (DBP), and heart rate (HR). Demographic and laboratory data were obtained for all patients from the hospital’s medical records to determine other associated cardiovascular risk factors, lipid profile, as well as renal and liver functions. The diagnosis of SH, chronic heart failure (CHF), chronic coronary syndrome (CCS), and the classification of the CVRP was realized based on patients’ medical history according to guideline recommendations [21,22]. DM-2 was diagnosed based on the medical history, basal blood glucose (BBG), and glycated hemoglobin levels. Cranial computed tomography was not considered mandatory for the diagnosis of CD. A transthoracic echocardiography (TTE) was performed in all patients to determine cardiac abnormalities. In patients with DM-2 and AF, we calculated the CHA_2_DS_2_-VASc score. In all patients, a rest electrocardiogram (ECG) was performed, and a Doppler carotid artery ultrasound was employed to evaluate the presence of atherosclerotic plaques and to determine intima-media thickness (IMT). An ankle−brachial index (ABI) was also assessed.

The presence and severity of CD were determined by a senior physician in neurology with the help of several scales that are widely employed in healthcare units and available free of charge in Romanian: Mini-Mental State Examination (MMSE), Montreal Cognitive Assessment (MoCA), Activities of Daily Living (ADL), Instrumental Activities of Daily Living (IADL), and the Geriatric Depression Scale GDS-15.

The study was conducted according to the guidelines of the Declaration of Helsinki and was approved by the Institutional Review Board of the Municipal Emergency Hospital Timisoara, Romania No. E1518/17.03.2021.

### 2.2. Clinical Assessment

#### 2.2.1. Parameters to Assess Subclinical Atherosclerosis

(a) IMT: A General Electric Vivid E9 ultrasound system was used to calculate the IMT value of all patients in the study. IMT was measured bilateral, at the level of the distal wall of the common carotid artery (CCA), 1 cm from the carotid bulb, with a 9 L MHz transducer. For each patient, 10 measurements were taken and average values were recorded.
(b) The ABI measurement is a non-invasive and quick test to verify the presence of peripheral artery disease (PAD). A score between 1.0 and 1.4 represents a normal ABI score, thus indicating no signs of PAD, while a score between 0.91 and 0.99 indicates borderline PAD, and a score lower than 0.9 designated the presence of PAD [23].

#### 2.2.2. Triglyceride-Glucose index (TyG)

The TyG is a reproducible, noninvasively, and accessible parameter for assessing IR that is independent of ongoing glucose-lowering medication [7]. It is calculated with the following formula: ln[BBG(mg/dL) × TG (mg/dL)/2], where TG means fasting triglyceride levels. TyG is considered a surrogate marker for IR, as it correlates with lipotoxicity and glucotoxicity [7,10]. A close connection has been demonstrated between TyG and DM-2, endothelial dysfunction, SH, CVD, and stroke [11,24] According to experts, the normal cut-off values of TyG vary widely between 4 and, due to the location of 2 in the TyG index formula [7,8,9].

#### 2.2.3. Neuropsychological Tests

(a) The Montreal Cognitive Assessment (MoCA) is a scale used to detect cognitive disorders. Its administration takes approximately 10 min, scores range between 0 and 30, and it analyzes several cognitive functions, such as orientation, language, visuospatial ability, working memory, short-term memory recall task, attention, concentration, executive functions, a two-item verbal abstraction task, a phonemic fluency task, and a three-dimensional cube copy. The MoCA is effective for the early identification of CD and signs of dementia. Values over 26 can be considered normal, while one under 26 indicates CD [25].
(b) The Mini-Mental State Examination (MMSE) is a scale with a duration of 10 min that is employed for the evaluation, detection, and quantification of CD, as well as for follow-ups. Its scores range between 0 and 30 points, and it analyzes several parameters of cognitive function (orientation, attention, recall, calculation, construction practices, and language manipulation). A score between 24 and 27 may represent an initial decrease in cognitive function compared to normal, while scores under 24 indicate the onset of dementia. MMSE examination is utilized for the screening of CD in regard to assessing its severity and progression, as well as to analyze a patient’s evolution. This score should be analyzed according to the level of education and age of the subject [26,27]; thirty is the maximum value, while scores equal to or lower than 24 are representative of dementia [26,28]. Additionally, a score between 24 and 27 means mild CD.
(c) Activities of daily living (ADL) is employed to evaluate the patient’s daily activities. Its application lasts approximately 10 min, and it analyzes 5 domains of the patient’s daily functional state (eating, personal hygiene, dressing, maintaining continence, and transferring/mobility). The score is between 0 (reduced functionality) and 10 (increased functionality or normal) [29,30].
(d) Instrumental Activities of Daily Living (IADL) is used to analyze the patient’s daily practical activities, an evaluation taking approximately 10 min. The IADL test involves an interview with the patient or a written questionnaire. It includes 8 more areas for evaluating the ability to function and daily care, such as shopping, cooking, or financial management, with a cumulative score ranging from 0, which represents low functionality, to 8, which represents high functionality [29,30].
(e) Geriatric Depression Scale (GDS-15) is a scale with 30 questions used to highlight the presence of depression among patients. It is a shorter version of the GDS, with 15 questions. Its administration takes about 10–15 min and is easier to apply in older patients or in those with CD, where a smaller number of questions is necessary [31,32]. Scores below 4 are considered normal, those between 5 and 8 indicate mild depression, scores between 9 and 11 indicate moderate depression, and scores between 12 and 15 indicate elements of severe depression. The obtained scores may provide information related to the presence or not of elements of depression and/or CD [32,33].

While each of these scales is commonly used to assess CD, each has its own strengths and limitations, and in order to minimize biases, we applied them all. The MMSE and MoCA scales are the most widespread neuropsychometric CD and dementia assessment tools; they complement each other and can be used for the screening, diagnosis, evaluation, and follow-up of cognitive disorders. To evaluate the patient’s self-care capacity, we applied the ADL and IADL scales. Patients with CD and associated CVD are prone to develop depression, which is why we employed the short version of the GDS-15 scale.

#### 2.2.4. Transthoracic Echocardiography (TTE)

TTE was performed according to guidelines recommendations [34] using a General Electric Vivid E9 ultrasound system with a M5S MHz transducer to evaluate the left ventricular (LV) and left atrial (LA) dimensions and functions.

We measured the standard diameters and volumes of cardiac structures and assessed LV performance by measuring the LV ejection fraction (LVEF), employing the modified Simpson rule. We determined the trans-mitral flow velocities in a pulsed Doppler from an apical window by placing the probe in a parallel alignment with the mitral blood flow to quantify the maximum velocity of the early filling wave (E-wave). To assess LA dimensions, we measured its diameter from a 4-chamber view in end-systole [34].

#### 2.2.5. CHA_2_DS_2_-VASc Score

The CHA_2_DS_2_-VASc score for assessing the AF Stroke Risk is used to evaluate the risk of stroke in patients with AF. The score matches the presence of the following pathologies to a number: CHF to 1, SH to 1, age equal to or over 75 years to 2, DM-2 to 1, stroke/thromboembolism to 2, PAD to 1, age between 65 and 74 years to 1, and female gender that confers a higher risk to 1. The higher the score, the greater the risk of stroke, and, therefore, oral anticoagulant therapy should be recommended from values over 1 in men and over 2 in women. The CHA2DS2-VASc score has been validated by many studies [35].

#### 2.2.6. Statistical Analysis

Results are expressed as percentages for categorical data and as mean value ± standard deviation for continuous data. Assessment of blood tests, HR, SBP, DBP, IMT, ABI, LV function parameters, memory, depression, and the activity and instrumental activity of daily living tests were undertaken using an impaired T-test in all three groups of patients. The evaluation by CVRF with/without DM-2, analysis regarding gender, age < 65 years old, or over 65 years old in CVRF and DM-2 patients, and analysis regarding the CHA_2_DS_2_-VASc score </>3 in patients with DM-2 and AF were determined by using the impaired T-test. Also, the Chi-squared test was used to examine differences between categorical variables. To evaluate the prognostic factors for CD, depending on CVRF, we employed the statistical method of logistic regression, considering a forward method based on the Wald test to determine the significant variables for which the odds ratios (OR) with corresponding 95% confidence intervals (CI) were reported. In addition, the final models were evaluated through sensitivity, specificity, positive predicted value (PPV), a negative predicted value (NPV), and an associated ROC curve with a corresponding area under the ROC curve. Statistical analysis was performed using IBM SPSS Statistics version 20.0 software for Windows with a significance level of 0.05.

Since a key outcome of our study was the MMSE values, we checked the corresponding achieved power for the MMSE values from the three subgroups of patients by using G*Power 3.1.9.2 software. We determined the achieved power between our 3 groups and obtained the following results: With Group A versus Group B, we obtained the effect size of 1.25 for the corresponding sample sizes, and the G*Power 3.1.9.2 software showed an achieved power of 99%. For Group A versus Group C the effect size was −1.29 and the calculated achieved power of 99%, while for Group B versus Group C the effect size was −2.54 and the achieved power was 100%. Our statistical analysis demonstrated that our sample size was large enough to draw statistically relevant conclusions, as a total power of over 80% is considered relevant.

## 3. Results

The 160 patients enrolled in our study had a mean age of 71.02 ± 9.98, ranging between 45 to 89 years. Among them, the female gender prevailed, as there were 86 (53.8%) women, and 74 (46, 3%) men. According to age groups, 48 (30%) subjects were younger than 65 years and 112 (70%) were older than 65 years.

According to our findings, patients in Group A with DM-2 were statistically younger compared to Group B, with DM-2 and FA (*p* = 0.041) (see Table 1). We did not find statistically significant differences between Groups A and B regarding SBP, DBP, and HR, nor referring to body mass index (BMI), although its values were significantly higher compared to the control group (*p* = 0.008, respectively *p* = 0.013) (see Table 1). Referring to the laboratory data, the parameters characterizing the lipid profile did not vary statistically significantly between Groups A and B, nor did we notice differences regarding BBG; however, although all patients had a TyG index in the normal range, patients from Group A had a significantly lower value compared to those from Group B (*p* = 0.012) (see Table 1).

In Group A, of 50 patients with DM-2, 45 subjects (90%) were diagnosed with systemic hypertension (SH) of various severities, as follows: 30% of patients had SH grade I, 42% grade II, and 18% grade III. CCS was present in 18 subjects (36%), without varying significantly from Groups B and C, while CHF was diagnosed in 17 subjects (34%), statistically significantly lower than Group B (*p* = 0.01). PAD had a low incidence in all three patient groups. There was no significant difference regarding the prevalence of stroke between the patients from our groups. CKD reached a high incidence in all three groups, being diagnosed in 64% of the subjects from Group A. In the two groups of patients with DM-2 (A and B), obesity was a common finding, being significantly more frequent than in Group C (*p* < 0.01). In Group A, 23 subjects (46%) were obese, while in Group C only 12 patients (21.4%) had an IMC of over 30. Regarding hyperlipemia and smoking, there were no significant differences between Groups A, B, and C (see Table 2).

In Group B, of subjects with DM-2 and AF, there were 49 individuals (over 90%) with SH, 16.7% of which with grade I, 37% with grade II, and 37% with grade III. CCS had the highest incidence in Group B, being diagnosed in 28 patients (52%). CHF was diagnosed in the majority of our patients, reaching the highest incidence in Group B, being present in 32 subjects (59.3%), statistically significantly more frequently compared to Group C (*p* = 0.036). In Group B, persistent AF prevailed, being encountered in 40% of patients, followed by permanent AF, which was present in 33% of them, and paroxysmal AF in 26%. It should be mentioned that all subjects from Group B were treated with new oral anticoagulants. In Group B, CKD was diagnosed in 72.2% of patients, statistically significantly higher than in Group C (*p* = 0.006). In Group B, 24 patients (44.5%) had an IMC of over 30 kg/m^2^, significantly higher than in Group C (*p* = 0.022), see Table 2.

In Group C, of patients without DM-2 or FA, only 30.4% of them had SH, while 14.3% had grade I, 9% had grade II, and 7% had grade III. CCS was encountered in 24 subjects (43%), and CHF in 22 (39.3%), mostly NYHA class I and II. In this group, CKD was present in 46.6% of patients (see Table 2).

As presented in Table 2, the majority of selected patients with SH also had clinical target organ damage, placing them in the very high-risk category. Even in subjects from Group C, without DM-2 and AF, these chronic cardiovascular conditions, CKD, and an impaired CVRP had a high prevalence. However, TTE and vascular sonography were performed in all subjects, as seen in Table 3. Regarding TTE parameters, patients from Group A had a significantly lower LA diameter compared to those from Group B (*p* = 0.029), and those from Group B had significantly higher LA diameters compared to Group C (*p* = 0.002). Although patients from Group B had higher LV end-diastolic and end-systolic diameters, there were no statistically significant differences compared to Group A. LVEF was the lowest in Group B (56.61% ± 7.43) compared to 58.32% ± 5.55 in Group C, but not statistically significant. Parameters estimating the right ventricular function, as the maximal tricuspid velocity (TRVmax), and estimated systolic pulmonary artery pressure (sPAP), were significantly lower in Group A compared to Group B (*p* = 0.05, respectively *p* = 0.029) and higher in Group B compared to Group C (*p* = 0.015, respectively *p* = 0.015), as seen in Table 3.

Regarding IMT values, the highest were determined in patients from Group B, seen to be significantly higher than in Group A, but not when compared to Group C, as seen in Table 3.

Concerning ABI measurements, there were no statistically significant differences between the patients from all three groups (Table 3).

The evaluation of CD by employing neuropsychological tests revealed that, although the values of MMSE, MoCA, ADL, IADL, and GDS-15 were more severely altered in patients from Group A and B versus Group C, we noticed a statistically significant difference only between Group B and C (see Table 4). We observed a statistically significant decrease in cognitive parameters (MMSE and MoCA (*p* < 0.05), as well as a reduction in those assessing the patients’ daily activity (ADL, IADL) (*p* < 0.05), as well as an augmentation of indexes assessing the intensity of depression (GDS-15) (*p* < 0.05) in patients from Groups A and B compared to subjects without DM-2 and/or AF (Group C), (see Table 4).

Considering all 160 patients evaluated in the study, 107 (66.9%) of them had MMSE scores over 27, while in 23, (14.4%) MMSE levels ranged between 24 and 26, indicating a slight CD, and 30 (18.8%) had MMSE scores lower than 24, defining the onset of dementia. Of the 104 patients with DM-2 evaluated in the study, 60 (57.7%) had MMSE scores above 27 (normal), 18 (17.3%) had scores between 24 and 26, signifying a slight CD, and 26 (25%) had MMSE scores under 24, indicating newly diagnosed dementia.

In Group B, 26 (48.1%) patients had MMSE scores above 27, 10 (18.5%) had scores between 24 and 26, indicating a mild CD, and 18 (33.3%) of them had MMSE scores under 24, characterizing dementia (see Table 4). This means that diabetic patients with associated AF may have more frequent and severe CD, slightly altered daily activity, and associated depression compared to those without DM-2.

In contrast, in Group C the prevalence of mild CD or incipient signs of dementia was lower. There were only 5 (8.9%) patients with MMSE scores between 24 and 26, 4 (7.1%) with MMSE under 24, while the remaining 47 (83.9%) subjects had an MMSE score over 27, being therefore considered without CD (Table 4).

To evaluate the prognostic factors for developing CD, quantified by an MMSE score under 27, and under 24 for dementia, in patients from Group A (with DM-2), Group B (DM-2 and AF), and Group A and B (DM-2 and DM-2 with AF), as well as to assess the OR, we employed logistic regression modeling to identify the most important predicting factors for these groups: age, gender, SH, smoking, hyperlipemia, obesity, and CKD. A forward method based on the Wald test was employed to determine the significant variables. The model was evaluated for sensitivity, specificity, PPV, NPV, and an associated ROC curve, with a corresponding area under the ROC curve. Logistic regression analysis revealed statistically significant higher odds for MMSE < 27 at an increased age when considering patients from Group A compared to Group B. In Group A, for an additional year in age, the odds for an MMSE < 27 was higher by a factor of 1.154 (OR = 1.154, 95%CI: 1.057; 1.259), while in Group B the odds for an MMSE < 27 was higher by a factor of 1.073 (OR = 1.073; 95%CI: 1.004; 1.146) (Table 5). In addition, statistically significantly higher odds for MMSE < 27 for increased age and hyperlipemia were identified when cumulating Groups, A and B (Table 1). For an additional year in age, the odds for an MMSE < 27 was higher by a factor of 1.123 (OR = 1.123; 95%CI: 1.061; 1.189), and patients with hyperlipemia had 3.946 times higher odds for MMSE < 27 versus subjects without hyperlipemia (OR = 3.946; 95%CI: 1.161; 7.475). Gender, smoking, hyperlipemia, obesity, CKD, and SH were not statistically significant in all patients with DM-2 independent of the presence of AF.

**Figure 1:**
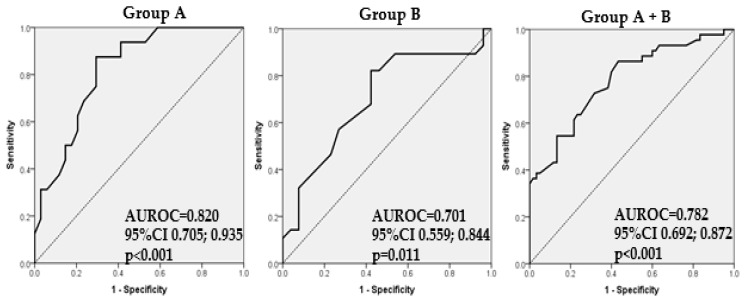
Receiver Operating Characteristic (ROC) curve for multiple logistic regression model with independent variables for the association of MMSE under 27 in patients with DM-2 (Group A) (AUROC = 0.820, 95%CI 0.705; 0.935, *p* < 0.001), in those with DM-2 and AF (Group B) (AUROC = 0.701, 95%CI 0.559; 0.844, *p* = 0.011), and for the association of MMSE lower than 27 for patients with DM-2 and patients with DM-2 and AF (AUROC = 0.782, 95%CI 0.692; 0.872, *p* < 0.001).

The probability of a decrease in MMSE scores under 27 for patients from Group A can be estimated by the formula exp(RS)/(1 + exp(RS)), where RS = −10.993 + 0.143 × (age). This model classified 74% of the patients correctly. The model had a sensitivity of 50.00%, specificity of 85.29%, PPV of 61.54%, and an NPV of 78.38%. The area under the ROC curve for this model (Figure 1) was 0.820 (AUROC = 0.820, 95%CI: 0.705; 0.935, *p* < 0.001).

In Group B the same probability can be estimated by the formula: exp(RS)/(1 + exp(RS)), where RS = −5.080 + 0.070 × (age). This model classified 68.52% of the patients correctly. The model had a sensitivity of 78.57%, a specificity of 57.69%, a PPV of 66.67%, and a NPV of 71.43 The area under the ROC curve for this model (Figure 1) was 0.701 (AUROC = 0.701, 95%CI: 0.559; 0.844, *p* = 0.011). In the cumulated population (Group A and B), the same probability can be estimated by the formula: exp(RS)/(1 + exp(RS)), where RS = 0.116+ 0.116 × (age) + 1.081 × (hyperlipemia), where hyperlipemia is 1 if the patient has hyperlipemia and 0 if the patient does not. This model classified 69.16% of the patients correctly. The model had a sensitivity of 57.45%, a specificity of 78.33%, a positive predicted value PPV of 67.50%, and a negative predicted value (NPV) of 70.15%. The area under the ROC curve for this model (Figure 1) was 0.782 (AUROC = 0.782, 95%CI: 0.692; 0.872, *p* < 0.001).

To evaluate the prognostic factors for developing severe CD, namely dementia, quantified by MMSE under 24, in patients from Group A (with DM-2), Group B (DM-2 and AF), and Group A and B (DM-2 and DM-2 with AF), and to assess the OR, we employed a logistic regression modeling for patients from these groups to identify the most important predicting factors: age, gender, hypertension, smoking, hyperlipemia, obesity, and CKD. The logistic regression analysis revealed a statistically significantly higher odds ratio or the risk of an MMSE score decrease under 24 parallel with advancing in age. Similarly, in Group B, statistically significantly higher odds for an MMSE score lower than 24 were noted in older patients (Table 6). Gender, hypertension smoking, hyperlipemia, obesity, and CKD did not prove to be statistically significant factors when considering subjects from Group A, Group B, or Groups A and B taken together.

**Figure 2:**
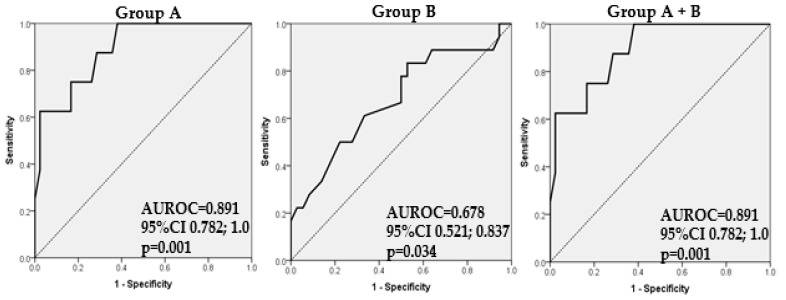
Receiver Operating Characteristic (ROC) curve for multiple logistic regression model with independent variables for the association of MMSE under 24 in patients with DM-2 (Group A) (AUROC = 0.891, 95%CI 0.782; 1.000, *p* = 0.001), in those with DM-2 and AF (Group B) (AUROC = 0.678, 95%CI 0.521; 0.836, *p* = 0.034) and in subjects from the conjoint Groups A and B (AUROC = 0.891, 95%CI 0.782; 1.000, *p* = 0.001).

The ROC curves measure the amount of separation between the distribution of predicted probabilities obtained by the final multiple logistic regression models in the diseased population from the distribution of the corresponding predicted probabilities in the non-diseased population, where ‘diseased’ population means an MMSE score under 27 in Figure 1 (for the specified set of groups of patients) and an MMSE score under 24 in Figure 2 (for the specified set of groups of patients).

Practically, for Figure 1, the AUC gives the probability that the calculated predicted probability obtained by the final multiple logistic regression model for a randomly chosen subject with MMSE under 27 exceeds that for a randomly chosen subject with an MMSE score above 27, and similarly in Figure 2 for an MMSE score under 24.

The statistical regression analyses revealed that, in patients from Group A, Group B, and those conjointly in Group A and B, the odds or risk of developing dementia, defined by a decrease in an MMSE score under 24, was higher for older patients. For an additional increase of one year in age, the odds of dementia were higher by a factor of 1.235 (OR = 1.235, 95%CI 1.069; 2.428) in Group A, and, in Group B, for any additional one-year increase in age, these odds were higher by a factor of 1.076 (OR = 1.076, 95%CI 0.999; 1.158) (thus borderline significant results) (*p* = 0.052), while, for those in Groups A and B conjointly, for every additional age year, the odds of dementia were higher by a factor of 1.128 (OR = 1.128, 95%CI 1.058; 1.202).

To determine the sensitivity, specificity, PPV, and NPV value for our patients’ groups, we generate an associated ROC curve corresponding to each logistic model (see Figure 2). The probability of a decrease in MMSE scores under 24 in patients from Group A may be estimated by the formula: exp(RS)/(1 + exp(RS)), where RS = −17.540 + 0.211 (age). This model classified 92% of the patients correctly. The model had a sensitivity of 62.50%, a specificity of 97.61%, a PPV of 83.33%, and an NPV of 93.18%. The area under the ROC curve for this model was 0.891 (AUROC = 0.891, 95%CI 0.782; 1.000, *p* < 0.001). In patients from Group B, the same probability can be estimated by the formula: exp(RS)/(1 + exp(RS)), where RS = −6.117+ 0.073x (age). This model classified 70.37% of patients correctly. The model had a sensitivity of 22.22%, specificity of 94.44%, a PPV of 66.67%, and an NPV of 70.83. The area under the ROC curve for this model (Figure 2 Group B) was 0.678 (AUROC = 0.678, 95%CI: 0.521; 0.836, *p* = 0.034). The probability of an MMSE score decrease under 24 in the conjoint group of patients with DM-2 and with DM-2 and AF can be estimated by the formula: exp(RS)/(1 + exp(RS)), where RS = −10.002+ 0.120x (age). This model classified 78.85% of patients correctly. The model had a sensitivity of 26.92%, a specificity of 96.15%, a PPV of 70.00%, and an NPV of 79.78%. The area under the ROC curve for this model was 0.891 (AUROC = 0.891, 95%CI: 0.782; 1.000, *p* = 0.001), as seen in Figure 2.

## 4. Discussion

The contribution of AF for developing CD and dementia represents a huge burden for society, especially in older patients, with AF being a major factor for stroke and also associated with CVD and general mortality. Other important cardiovascular risk factors, such as SH, DM-2, dyslipidemia, obesity, and changes in renal function, are also contributing to the occurrence of CD; therefore, this association can be multifactorial, with complex associated pathophysiological pathways.

Several mechanisms have been proposed for the association between AF and cognitive impairment in several clinical studies. The main factor of AF-induced cognitive impairment appears to be cerebral infarction, but there are other mechanisms to consider, including silent cerebral infarctions, AF-related cerebral hypoperfusion, inflammation, microhemorrhage and cerebral atrophy, or systemic atherosclerotic vascular disease [17]. Some studies specify the fact that, to detect more precisely the link between AF and dementia, the evaluation of biological biomarkers (from cerebrospinal fluid or plasma) and brain imaging associated with cognitive impairment tests should be recommended in the population with AF. In the past decade, much progress has been made in detecting biomarkers with diagnostic and prognostic value for dementia [36]. Identification of specific biomarkers that predict cognitive decline in patients with AF could aid in screening and management strategies, and biomarkers may be useful in risk stratification in patients considered clinically low risk. However, the limitations of biomarkers for everyday clinical use must balance predictive ability with practicality [37]. DM-2 can affect neuronal and cognitive function through hypoperfusion of brain tissues caused by cerebrovascular diseases, changes in glucose transporters that cause abnormalities in the uptake and neuronal metabolism of glucose, IR, recurrent hypoglycemic episodes of diabetes pharmacotherapy, and local hyper- and hypometabolism of brain areas, which result in neuronal damage [38]. In patients with DM-2 and CD, a thorough assessment of cerebral metabolic characteristics and their impact on cognition is particularly important for their management. Understanding the relationship between DM-2 and cognitive function is necessary to enable physicians to manage their patients in everyday clinical practice.

In the medical literature, several studies have debated the association between DM-2 and the occurrence of CD and dementia. CD may be present at any age, albeit mostly in older patients, considering that cognitive impairment extends over a longer period. It has been discussed that metabolic dysfunctions may contribute to an earlier onset of CD symptoms. In a recent review [39], the mechanisms responsible for the occurrence of CD in association with DM-2 were evaluated in diabetic mice, and it has been suggested that increased levels of pro-inflammatory cytokines, diabetes, and obesity may cause decreased spatial recognition memory, sustaining a relationship between cognitive loss and inflammation. The pathogenesis of some neurodegenerative diseases and diabetes includes mitochondrial dysfunction, which is attributed to the production of reactive oxygen species, indicating a relationship between neuroinflammation and ROS in cognitive impairment [39].

In a Swedish study [40] conducted on 2746 patients aged over 60 years, for a period of 9 years, the effects of DM-2 and prediabetes on CD were evaluated by analyzing the patients’ MMSE scores. Brain magnetic resonance imaging performed in 455 patients showed that prediabetes and DM-2 were independently associated with accelerated CD compared to subjects without DM-2 (*p* < 0.01), leading to the conclusion that DM-2 and prediabetes might predispose to microvascular lesions and accelerated CD.

In another recent study that evaluated 332 patients with DM-2 with an average duration of DM- of 10.17 ± 4.81 years, following each patient for over one year, the MMSE score was assessed to confirm the presence or absence of CD, and 81 of them (24.4%) were diagnosed with cognitive impairment, with a statistically significant difference in MMSE between patients under 60 years old compared to those over 60 years old and in those with a longer duration of DM-2 (*p* < 0.001). The conclusion was that CD is frequent among patients with DM-2, independent of gender, and is directly related to age and the duration of diabetes [41]. In another study conducted on 208 patients, 80 with DM-2 and 128 controls, the ADL and IADL scores were lower in diabetic subjects than in controls (*p* < 0.05). These findings and multiple DM-2-related complications were associated with CD in older diabetic patients [42].

A study carried out in India that evaluated the presence of CD in diabetic patients by evaluating the MoCA concluded that its scores were statistically significantly lower in patients with DM-2 compared to those without (*p* < 0.001). In patients with DM-2, MoCA assessment indicated the significant influence of age on CD (*p* < 0.001) [43]. Another study following 3687 participants over 6 years, of whom 6.4% had DM-2, indicated that both MMSE and MoCA scores were lower in patients with DM-2 compared to those without. The initial evaluations showed a decrease in MMSE and MoCA values related to age, male sex, as well as the presence of DM-2, SH, and stroke. After 6 years of follow-up, MMSE changes were greater in patients with DM-2, indicating an increased risk for CD [44]. Similarly, another study that compared 100 diabetic patients with 100 non-DM-2 controls, highlighted significantly lower MoCA scores among the first group (*p* < 0.001). The rate of mild CD was significantly higher among DM-2 patients compared to controls (*p* < 0.01, and OR = 2.8 [95%CI: 1.2–6.5]), and also in patients with SH compared to non-hypertensive ones, as well as in subjects aged over 50 years compared to younger ones as well (*p* < 0.05). In diabetic patients aged over 50 years, age was the only significant predicting factor for mild CD (OR = 2.9 [95%CI: 3.8–123.3], *p* < 0.001). The study concluded that patients with DM-2 have a significant three times higher risk of developing mild CD compared to non-diabetic subjects. Age, CVD, SH, frequency of hypoglycemic episodes, and duration of diabetes were risk factors for cognitive impairment [45]. In a meta-analysis that assessed the risk of CD and its progression to dementia in DM-2 patients with/or without depression, it was shown that the presence of depression in patients with DM-2 increases the risk of CD and dementia (HR = 1.82; [1.79, 1.85]) compared to DM-2 patients without depression [46]. In another study [47] the presence of undiagnosed depression, assessed by GDS-15, in patients with DM-2 was not unusual, being present in 13% of them. Patients with depression frequently had a prior history of comorbidities, tended to be more obese, had a higher SBP (*p* = 0.01), with similar control in DBP and blood lipid values, had higher BBG and glycated hemoglobin, as well as more frequent self-reported hypoglycemic events (*p* = 0.03) and longer disease durations (*p* = 0.02). Thus, in elderly patients with DM-2, depression was present in those with episodes of hypoglycemia, longer disease duration, and associated comorbidities, such as endothelial dysfunction and other risk factors, as also reported in some studies [47,48].

In our research, we also documented a higher prevalence of CVD in patients with DM-2, especially in those with associated AF. Diabetic patients had significantly more altered MMSE, MoCA, ADL, IADL, and GDS-15 scores compared to subjects with a very high CVRP but without DM-2. As presented in some of the above-mentioned studies [41,45], aging represents a determinant risk factor for CD and dementia as well (OR = 1.128 [95%CI: 1.058–1.202], *p* = 0.037). In our study, the statistical analysis also confirmed that age has an important impact on cognitive function, assessed by the MMSE scores of patients with DM-2 independently of AF and other risk factors.

Several recent studies have highlighted the considerable contribution of AF for the development of CD [15,49]. In a study published in 2023 by Steffen Blum et al., it has been highlighted that patients with AF have a 1.5 times higher risk of CD compared to the general population and, after an ischemic stroke, this risk augments by three times [15]. A large cohort study, the “Atherosclerosis Risk in Communities Neurocognitive Study”, followed up 12.515 middle-aged participants for 24 years and determined that 16.82% of them developed AF, while 9.24% developed dementia, with AF being associated with a significantly greater decline in assessment scores except for the Delayed Word Recall Test. This study concluded that AF was associated with greater CD and increased risk of dementia, independent to the presence of ischemic stroke [49]. Similarly, in our study, we intended to analyze the additional impact of metabolic disturbances, especially DM-2, and we determined that the association of DM-2 and AF increases the risk for CD in comparison to DM-2 alone. In our study, by using five neuropsychological tests, we have documented a more severe cognitive impairment in patients with AF independently to the presence of DM-2, but the difference was not statistically significant. This observation was also confirmed by the multivariate regression analysis. A study that evaluated the incidence of mild CD or dementia in a group of 2577 participants aged over 60 years with two or more cardiovascular and/or metabolic disorders (DM-2, CCS, or stroke) (following them for 12 years) demonstrated that the association of multiple cardiometabolic pathologies increases the risk of mild CD (HR = 1.73; 95%CI: 1.23; 2.44) and the progression to dementia (HR = 1.86; 95%CI: 1.17; 2.97) [5].

Another study that evaluated the presence or absence of CD, as assessed by MoCA scores, in 70 patients with DM-2 in relation to the lipid profile (total cholesterol, high-density lipoprotein, low-density lipoprotein, and triglycerides) failed to demonstrate any statistically significant direct association between them [50]. In contrast, in a study that evaluated 290 patients with DM-2, it was observed that elevated LDL cholesterol was a risk factor for mild CD (OR = 1.635, *p* = 0.047), concluding that mild CD is frequent among DM-2 patients [51]. On the other hand, the finding from another study showed no correlation between the levels of total cholesterol, LDL-cholesterol, HDL-cholesterol, and TG and apolipoprotein B and CD, except for apolipoprotein A1 levels, which were independently associated with CD (OR = 5.201, *p* = 0.024) and correlated negatively to significant MMSE scores (r = −0.132, *p* = 0.016) and MoCA scores (r = −0.143, *p* = 0.009) [52]. In our study, we also demonstrated that, in diabetic patients, the presence of hyperlipidemia increased the risk of a reduced MMSE score, and thus of developing CD or dementia.

Regarding the assessment of macrovascular complications, it is recommended that the carotid IMT should be included in the routine screening of vascular impairments in patients with prediabetes and newly diagnosed DM-2. It was demonstrated that IMT values were increased among patients with DM-2 compared to those with prediabetes and to those with normal FPG. The values of SBP, DBP, FPG, total and LDL-cholesterol, TG, and serum creatinine were also higher in patients with DM-2 and prediabetes than in controls [53]. Similarly, another study that evaluated 142 patients with DM-2 and CKD over 7 years analyzed whether the IMT represents an independent prognostic factor for cardiovascular morbidity and mortality, concluding that its values represent a strong and independent predictor of CV morbidity and mortality [54]. PAD is one of the major long-term complications of diabetes. The benefit of ABI measurement was evaluated in 85 participants, of which 40 had DM-2, and it was highlighted that this assessment was slightly less reliable in diabetic people than in those without [55]. In contrast, a study carried out on 48 patients with DM-2 evidenced reduced values of ABI in half of them, with the prevalence of PAD being approximately 56.25% in patients with DM-2 [56]. The assessment of IMT and ABI as parameters of vascular remodeling were also analyzed in connection with other diseases, even with some specific therapies [57].

The significant contribution of DM-2 for developing mild CD in patients with CVD, such as SH, CID, CHF, and especially with AF, was largely debated in the study of Dove et al., who identified DM-2 as an important risk factor for CD (HR = 1.34, 95%CI: 0.97–1.85, *p* = 0.08), even in the absence of a risk of progression to dementia. Patients who had glycated hemoglobin levels ≥7.5% had a 2-fold higher risk of mild CD (HR = 2.01) and a 3-fold higher progression from mild CD to dementia (HR = 2.87). The presence of CVD, associated with DM-2, doubled the risk of mild CD and its progression to dementia, being a multiplicative interaction between DM-2 and CVD for CD and dementia (*p* < 0.05) [5]. A more recent multicentric study, conducted on 7207 elderly women, evaluated annually the evolution of cognitive function and concluded that uncontrolled SH may lead to mild CD or even dementia, while also finding that adequate control of SH could reduce this risk [58].

A presence of approximately 9.5% of AF was highlighted in the Atherosclerosis Risk in Communities Neurocognitive Study (ARIC-NCS), which analyzed the presence of cognitive impairment in 6432 participants. In patients with AF, the prevalence of mild CD/Alzheimer’s disease, respectively vascular dementia was higher than in patients without AF, thus indicating an increased risk for CD (OR = 1.28; 95%CI: 1.04–1.562) and dementia (OR = 2.5; 95%CI: 1.64–3.10) [16]. Several mechanisms of the association between AF and CD have been identified. In addition to pro-inflammatory disorders and changes in cerebral blood flow, there are also brain microlesions or silent cerebral ischemia. Through changes in blood flow and cardiac rhythm, as well as of blood pressure, AF can lead to transient or chronic cerebral hypoperfusion and, potentially, to CD [17].

In a study that assessed 4593 participants with AF at baseline, an approximately 3.43-fold increased risk of mild CD in women and 1.73 in men has been identified. Similarly, the risk of dementia in patients with AF was also increased. After a follow-up period of over 4 years, approximately 30% of all patients progressed from normal cognition to mild CD, and 21% developed dementia. AF female patients had a statistically significantly higher risk of disease progression (HR = 1.21; [1.04, 1, 40]) compared to those without AF, and AF was associated with a statistically significant risk of developing mild CD or even vascular dementia [59]. Another study that evaluated the presence of CD and subclinical atherosclerosis in 155 patients with SH, of which 84 also had AF, highlighted that MMSE, MoCA, and LVEF scores were significantly decreased, while the GDS-15 and IMT amounts were significantly increased in patients with SH and AF compared to those without AF (*p* < 0.05). In patients with AF and CHA2DS2 –VASc > 3 aged over 65 years, MMSE scores were significantly lower, and IMT was significantly increased (*p* < 0.05). This study showed that cognitive impairment is found in hypertensive patients with AF, also indicating that there is a direct relationship between CD, depression, SH, AF, age, CHA2DS2-VASc score, IMT, and LVEF, with these evaluations being recommended for the prevention of CD in these patients and AF [60]. Another study suggests that there is a strong relationship between PAD and AF. The presence of PAD was associated with a 31% increase in the risk of incident AF, and an ABI >1.40 was not associated with an increased risk of AF, while an ABI <1.00 indicated an increased risk of developing AF (HR = 1.32; 95%CI: 1.18–1.47) [61].

Other studies analyzed the relationship between CD, DM-2, and CKD. Thus, a study carried out on 3399 patients with DM-2 showed that obesity and CD are independent risk factors for the progression of CKD, especially in women [62]. Otherwise, another study determined that patients with DM-2 and CKD, especially those with advanced kidney damage, may have cognitive disorders [63].

Considering the alarming increase in the prevalence of CVD and DM-2 worldwide, it seems that they will continue to represent major public health problems in the coming years. Detecting associated cognitive disorders, mental health issues, or changes in the care and daily activities among patients with DM-2 and AF as soon as possible may have clinical implications and allow adequate strategies for optimal therapeutic management of these patients, resulting in an improved evolution and more accurate approach to the associated comorbidities and any possible complications that may arise. More accurate evaluations of associated cognitive disorders and depression can be beneficial for developing adequate strategies for the prevention, treatment, and monitoring of the evolution of cognitive disorders, and also of other clinical, psychological, and therapeutic interventions to slow down the progress of these cognitive disorders that may affect the quality of life of patients and the accentuation of the degree of dementia.

Study limitation: Our study has several limitations, some of which we plan to address in future studies. Potentially the most important limitation is that it is a single-center study conducted on a limited population, of which 104 patients were afflicted with DM-2 and 54 with AF. Another limitation is the fact that we did not follow up on our patients, this being the objective of a future study. A third limitation is that we did not include a group of non-diabetic patients with AF in our study group, as large cohort studies have already pointed out that AF is an independent risk factor for CD [49] and, in our study, we analyzed the association between DM-2 and AF in comparison to patients with DM-2 alone. Although it is known that several cardio-metabolic pathologies represent individual risk factors for CD, their combined impact on the early onset and evolution of CD needs to be further studied. Thus, it is particularly important to continue research regarding patients with AF and/or DM-2 and to diagnose CD and dementia, as well as to increase the level of awareness of medical staff and patients regarding the early detection and prevention of cognitive disorders, identifying specific priorities and developing clinical, therapeutic, psychological and management strategies.

## 5. Conclusions

Our finding demonstrated that MMSE, MoCA, ADL, IADL, and GDS-15 scores are more severely altered in patients with DM-2, especially when associated with AF versus those without DM-2 and AF. Multivariate regression models have suggested that older age and the presence of dyslipidemia represent independent risk factors for the development of CD in patients with DM-2. Future studies should focus on researching the pathophysiological background of CD in patients with cardio-metabolic disorders as well as elaborating on new strategies for its early detection to preserve cognitive function in patients with DM-2 and AF, thus reducing the progression to dementia in those with functional affected cognition.

## Figures and Tables

**Table 1:** Clinical and laboratory data in all three groups.

Patients Demographic Data	Group A—50 P with DM-2	Group B—54 P with DM-2 and AF	Group C—56 Controls	*p* between A–B	*p* between A–C	*p* between B–C
Mean age	69.42 ± 10.39	73.44 ± 9.39	70.13 ± 9.91	0.041	0.722	0.074
Sex: male	28 (56.0%)	24 (44.4%)	22(39.3%)	0.239	0.085	0.583
female	22 (44.0%)	30 (55.6%)	34(60.7%)			
BMI (kg/m^2^)	32.40 ± 7.47	32.11 ± 7.29	28.56 ± 7.47	0.840	0.008	0.013
WC (cm)	95.60 ± 10.73	96.85 ± 9.83	91.20 ± 11.63	0.536	0.046	0.07
SBP (mmHg)	132.80 ± 20.75	134.63 ± 23.00	127.50 ± 15.10	0.672	0.133	0.059
DBP (mmHg)	78.50 ± 13.25	80.93 ± 14.73	73.93 ± 14.60	0.381	0.096	0.014
HR (b/min)	75.62 ± 13.88	78.13 ± 18.60	70.54 ± 11.89	0.440	0.045	0.013
Laboratory data						
Total chol. (mg/dL)	187.54 ± 55.04	175.96 ± 60.74	189.89 ± 64.70	0.312	0.842	0.247
LDL chol (mg/dL)	128.62 ± 49.35	129.33 ± 54.66	137.19 ± 53.76	0.945	0.396	0.449
HDL chol. (mg/dL)	50.86 ± 24.89	44.31 ± 11.37	49.67 ± 15.80	0.084	0.769	0.044
Triglycerides (mg/dL)	131.08 ± 58.05	128.66 ± 50.70	135.51 ± 62.39	0.821	0.706	0.530
Uric acid (mg/dL)	6.07 ± 1.61	6.37 ± 1.34	6.29 ± 1.72	0.311	0.512	0.783
Basal glycemia (mg/dL)	111.58 ± 24.07	115.70 ± 42.52	107.03 ± 17.52	0.549	0.266	0.162
eGRF (ml/min)	55.22 ± 14.53	52.88 ± 14.16	64.83 ± 19.24	0.408	0.004	0.000
TyG index	4.03 ± 1.65	4.67 ± 0.63	4.43 ± 1.16	0.012	0.159	0.174

Legend: P—patient; DM-2—diabetes mellitus type 2; AF—atrial fibrillation; BMI—body mass index; WC—waist circumference; SBP—systolic blood pressure; DBP—diastolic blood pressure; HR—heart rate; LDL chol.—Low-density lipoprotein; HDL chol. = High-density lipoprotein; eGRF—estimated glomerular filtration rate; TyG—triglyceride glucose index.

**Table 2:** Prevalence of SH, clinical organ damage, and risk factors in study groups.

Cardiovascular Pathology and Risk Factors	Group A—50 P with DM-2	Group B—54 P with DM-2 and AF	Group C—56 Controls	*p* between A–B	*p* between A–C	*p* between B–C
SH	45–90%	49–90.7%	17–30.4%	0.898	0.000	0.000
CCS	18–36%	28–51.9%	24–42.9%	0.104	0.471	0.345
CHF	17–34%	32–59.3%	22–39.3%	0.010	0.573	0.036
NYHA I	2–4%	2–3.7%	7–12.5%	0.937	0.117	0.092
NYHA II	11–22%	25–46.3%	10–17.9%	0.009	0.593	0.001
NYHA III	2–4%	4–7.4%	5–8.9%	0.457	0.308	0.771
NYHA IV	2–4%	1–1.9%	1–1.8%	0.513	0.493	0.979
PAD	5–10%	2–3.7%	3–5.4%	0.200	0.366	0.677
Lacunar stroke	7–14%	8–14.8%	11–19.6%	0.906	0.440	0.503
Minor stroke	4–8%	7–13%	6–10.7%	0.411	0.633	0.715
CKD	32–64%	39–72.2%	26–46.4%	0.368	0.070	0.006
CKD stage I	8–16%	8–14.8%	8–14.3%	0.867	0.806	0.937
CKD stage II	9–18%	12–22.2%	9–16.1%	0.592	0.792	0.412
CKD stage III	12–24%	14–25.9%	8–14.3%	0.821	0.202	0.127
CKD stage IV	3–6%	5–9.3%	1–1.8%	0.533	0.256	0.084
Obesity	23–46%	24–44.4%	12–21.4%	0.802	0.022	0.010
Obesity gr I	9–18%	9–16.7%	2–3.6%	0.857	0.015	0.022
Obesity gr II	6–12%	5–9.3%	4–7.1%	0.650	0.393	0.686
Obesity gr III	8–16%	10–18.5%	6–10.7%	0.564	0.422	0.161
Hyperlipemia	23–46%	33–61.1%	29–51.8%	0.122	0.552	0.324
Smoking	17–34%	13–24.1%	22–39.3%	0.264	0.573	0.087

Legend: P—patient; DM-2—diabetes mellitus type 2; AF—atrial fibrillation; SH—systemic hypertension; CCS—chronic coronary syndrome; CHF—chronic heart failure; NYHA—New York Heart Association; PAD—peripheral artery disease; CKD—chronic kidney disease.

**Table 3:** TTE and other ultrasonographic parameters in study groups.

Parameters	Group A—50 P with DM-2	Group B—54 P with DM-2 and AF	Group C—56 P without DM-2 and AF	*p* between A–B	*p* between A–C	*p* between B–C
TTE parameters						
LA (mm)	40.58 ± 6.00	43.40 ± 6.93	39.73 ± 5.15	0.029	0.436	0.002
IVS (mm)	11.99 ± 1.97	12.08 ± 1.89	11.82 ± 2.48	0.807	0.696	0.531
LVPW (mm)	12.16 ± 2.62	11.72 ± 2.07	12.39 ± 2.83	0.345	0.668	0.161
LVESD (mm)	23.40 ± 6.28	24.81 ± 7.29	24.39 ± 6.39	0.294	0.423	0.747
LVEDD (mm)	45.12 ± 4.84	46.88 ± 6.65	44.80 ± 3.67	0.127	0.668	0.046
LVESV (mL)	34.68 ± 15.06	38.90 ± 13.96	33.87 ± 12.10	0.141	0.761	0.046
LVEDV (mL)	74.14 ± 15.75	77.42 ± 16.54	71.37 ± 15.96	0.303	0.373	0.054
LVEF (%)	57.06 ± 7.14	56.20 ± 7.72	58.32 ± 5.55	0.560	0.310	0.101
E (m/s)	0.72 ± 0.15	0.74 ± 0.21	0.68 ± 0.19	0.650	0.328	0.191
TRVmax (m/s)	2.31 ± 0.41	2.50 ± 0.59	2.26 ± 0.0.40	0.050	0.595	0.015
sPAP (mmHg)	36.50 ± 10.81	41.68 ± 12.89	36.00 ± 11.11	0.029	0.815	0.015
Ultrasonographic parameters						
IMT (mm) left	0.63 ± 0.31	0.76 ± 0.26	0.68 ± 0.23	0.026	0.349	0.103
IMT (mm) right	0.62 ± 0.33	0.72 ± 0.29	0.66 ± 0.27	0.117	0.485	0.311
ABI left	1.12 ± 0.20	1.06 ± 0.10	1.10 ± 0.10	0.085	0.508	0.101
ABI right	1.07 ± 0.08	1.12 ± 0.19	1.10 ± 0.10	0.084	0.056	0.619

IVS: interventricular septum; LA: left atrial; LVPW: Left ventricular posterior wall; LVESD: left ventricular end-systolic diameter; LVEDD: left ventricular end-diastolic diameter; LVESV: left ventricular end systolic-volume; LVEDV: left ventricular end-diastolic volume; LVEF: left ventricular ejection fraction; E: peak early diastolic transmitral flow velocity; TRVmax: maximal tricuspid velocity; sPAP: pulmonary systolic arterial pressure; IMT: Intima-Media Thickness; ABI: Ankle Brachial Index.

**Table 4:** Neuropsychological tests in patients’ groups.

Neuropsychological Tests	Group A—50 P with DM-2	Group B—54 P with DM-2 and AF	Group C—56 Controls	*p* between A–B	*p* between A–C	*p* between B–C
MMSE	26.34 ± 4.48	25.09 ± 4.79	27.63 ± 3.68	0.175	0.109	0.003
MoCA	23.32 ± 5.16	22.89 ± 5.25	25.07 ± 4.67	0.674	0.069	0.023
ADL	9.40 ± 1.01	9.24 ± 1.14	9.66 ± 0.88	0.456	0.158	0.034
IADL	6.60 ± 1.86	6.56 ± 1.77	7.21 ± 1.38	0.901	0.060	0.033
GDS-15	6.88 ± 2.28	7.12 ± 2.38	6.23 ± 2.15	0.588	0.136	0.041

Legend: MMSE—Mini Mental State Examination Scale; MoCA—Montreal Cognitive Assessment Scale; ADL—Activities of Daily Living Score; IADL—Instrumental Activities of Daily Living Score; GDS-15—Geriatric Depression Scale 15 questions.

**Table 5:** Multiple logistic regression analysis for risk of MMSE < 27.

	Patients with MMSE < 27 *n* (%) *	OR (95%CI)	*p*-Value
Group A—Patients with DM-2			
Age (years)	16 (32.00%)	1.154 (1.057; 1.259)	0.001
Group B—Patients with DM-2 and AF			
Age (years)	28 (51.85%)	1.073 (1.004; 1.146)	0.037
Group A and B Patients with DM-2 and patients with DM-2 and AF			
Age (years)	44 (42.31%)	1.123 (1.061; 1.189)	<0.001
Hyperlipemia: No	15 (14.42%)	1	
Yes	29 (27.88%)	3.946 (1.161; 7.475)	0.030

Note: *n*= number of patients, OR = odds ratio, 95%CI = 95% confidence interval. * Percentages based on the total number of patients with DM-2 (50 patients), DM-2 and AF patients (54 patients), and all patients with DM-2 or DM-2 and AF (104 patients).

**Table 6:** Multiple logistic regression analysis for risk of MMSE < 24.

	Patients with MMSE < 24 *n* (%) *	OR (95%CI)	*p*-Value
Patients with DM-2			
Age (years)	8 (16.00%)	1.235 (1.069; 1.428)	0.004
Patients with DM-2 and AF			
Age (years)	18 (33.33%)	1.076 (0.999; 1.158)	0.052
Patients with DM-2 and Patients with DM-2 and AF			
Age (years)	26 (25.00%)	1.128 (1.058; 1.202)	0.037

Note: *n* = number of patients, OR = odds ratio, 95%CI = 95% confidence interval. * Percentages based on the total number of patients with DM-2 (50 patients), DM-2 and AF patients (54 patients), and all patients with DM-2 or DM-2 and AF (104 patients).

## Data Availability

The data supporting reported results can be obtained from the first author of this manuscript, Marius Militaru, upon request.

## References

[1] Brito D.V.C., Esteves F., Rajado A.T., Silva N., Araújo I., Bragança J., Castelo-Branco P., Nóbrega C. (2023). Assessing Cognitive Decline in the Aging Brain: Lessons from Rodent and Human Studies. NPJ Aging.

[2] Stieger M., Lachman M.E. (2021). Increases in Cognitive Activity Reduce Aging-Related Declines in Executive Functioning. Front. Psychiatry.

[3] Koh Y.H., Lew L.Z.W., Franke K.B., Elliott A.D., Lau D.H., Thiyagarajah A., Linz D., Arstall M., Tully P.J., Baune B.T. (2022). Predictive Role of Atrial Fibrillation in Cognitive Decline: A Systematic Review and Meta-Analysis of 2.8 Million Individuals. EP Eur..

[4] Varghese S.M., Joy N., John A.M., George G., Chandy G.M., Benjamin A.I. (2022). Sweet Memories or Not? A Comparative Study on Cognitive Impairment in Diabetes Mellitus. Front. Public Health.

[5] Dove A., Shang Y., Xu W., Grande G., Laukka E.J., Fratiglioni L., Marseglia A. (2021). The Impact of Diabetes on Cognitive Impairment and Its Progression to Dementia. Alzheimer’s Dement..

[6] Standl E., Khunti K., Hansen T.B., Schnell O. (2019). The Global Epidemics of Diabetes in the 21st Century: Current Situation and Perspectives. Eur. J. Prev. Cardiol..

[7] Lopez-Jaramillo P., Gomez-Arbelaez D., Martinez-Bello D., Abat M.E.M., Alhabib K.F., Avezum Á., Barbarash O., Chifamba J., Diaz M.L., Gulec S. (2023). Association of the Triglyceride Glucose Index as a Measure of Insulin Resistance with Mortality and Cardiovascular Disease in Populations from Five Continents (PURE Study): A Prospective Cohort Study. Lancet Healthy Longev..

[8] Tong X.-W., Zhang Y.-T., Yu Z.-W., Pu S.-D., Li X., Xu Y.-X., Shan Y.-Y., Gao X.-Y. (2022). Triglyceride Glucose Index Is Related with the Risk of Mild Cognitive Impairment in Type 2 Diabetes. DMSO.

[9] Weyman-Vela Y., Simental-Mendía L.E., Camacho-Luis A., Gamboa-Gómez C.I., Guerrero-Romero F. (2022). The Triglycerides and Glucose Index Is Associated with Mild Cognitive Impairment in Older Adults. Endocr. Res..

[10] Wang H., Ling Q., Wu Y., Zhang M. (2023). Association between the Triglyceride Glucose Index and Cognitive Impairment and Dementia: A Meta-Analysis. Front. Aging Neurosci..

[11] Liu X., He G., Lo K., Huang Y., Feng Y. (2021). The Triglyceride-Glucose Index, an Insulin Resistance Marker, was Non-Linear Associated with All-Cause and Cardiovascular Mortality in the General Population. Front. Cardiovasc. Med..

[12] Manolis T.A., Manolis A.A., Apostolopoulos E.J., Melita H., Manolis A.S. (2020). Atrial Fibrillation and Cognitive Impairment: An Associated Burden or Burden by Association?. Angiology.

[13] Sepehri Shamloo A., Dagres N., Müssigbrodt A., Stauber A., Kircher S., Richter S., Dinov B., Bertagnolli L., Husser-Bollmann D., Bollmann A. (2020). Atrial Fibrillation and Cognitive Impairment: New Insights and Future Directions. Heart Lung Circ..

[14] Madhavan M., Graff-Radford J., Piccini J.P., Gersh B.J. (2018). Cognitive Dysfunction in Atrial Fibrillation. Nat. Rev. Cardiol..

[15] Blum S., Conen D. (2023). Mechanisms and Clinical Manifestations of Cognitive Decline in Atrial Fibrillation Patients: Potential Implications for Preventing Dementia. Can. J. Cardiol..

[16] Alonso A., Knopman D.S., Gottesman R.F., Soliman E.Z., Shah A.J., O’Neal W.T., Norby F.L., Mosley T.H., Chen L.Y. (2017). Correlates of Dementia and Mild Cognitive Impairment in Patients with Atrial Fibrillation: The Atherosclerosis Risk in Communities Neurocognitive Study (ARIC-NCS). J. Am. Heart Assoc..

[17] Rivard L., Friberg L., Conen D., Healey J.S., Berge T., Boriani G., Brandes A., Calkins H., Camm A.J., Yee Chen L. (2022). Atrial Fibrillation and Dementia: A Report from the AF-SCREEN International Collaboration. Circulation.

[18] Atrial Fibrillation Diagnosis Associated with 45% Increased Risk of Mild Cognitive Impairment. https://www.news-medical.net/news/20231025/Atrial-fibrillation-diagnosis-associated-with-4525-increased-risk-of-mild-cognitive-impairment.aspx.

[19] Lippi G., Sanchis-Gomar F., Cervellin G. (2021). Global Epidemiology of Atrial Fibrillation: An Increasing Epidemic and Public Health Challenge. Int. J. Stroke.

[20] Khan M.A.B., Hashim M.J., King J.K., Govender R.D., Mustafa H., Al Kaabi J. (2020). Epidemiology of Type 2 Diabetes—Global Burden of Disease and Forecasted Trends. J. Epidemiol. Glob. Health.

[21] McDonagh T.A., Metra M., Adamo M., Gardner R.S., Baumbach A., Böhm M., Burri H., Butler J., Čelutkienė J., Chioncel O. (2021). 2021 ESC Guidelines for the Diagnosis and Treatment of Acute and Chronic Heart Failure. Eur. Heart J..

[22] Williams B., Mancia G., Spiering W., Agabiti Rosei E., Azizi M., Burnier M., Clement D.L., Coca A., de Simone G., Dominiczak A. (2018). 2018 ESC/ESH Guidelines for the Management of Arterial Hypertension: The Task Force for the Management of Arterial Hypertension of the European Society of Cardiology (ESC) and the European Society of Hypertension (ESH). Eur. Heart J..

[23] Le Bivic L., Magne J., Guy-Moyat B., Wojtyna H., Lacroix P., Blossier J.-D., Le Guyader A., Desormais I., Aboyans V. (2019). The Intrinsic Prognostic Value of the Ankle-Brachial Index Is Independent from Its Mode of Calculation. Vasc. Med..

[24] Li S., Deng X., Zhang Y. (2022). The Triglyceride-Glucose Index Is Associated with Longitudinal Cognitive Decline in a Middle-Aged to Elderly Population: A Cohort Study. J. Clin. Med..

[25] Dautzenberg G., Lijmer J., Beekman A. (2020). Diagnostic Accuracy of the Montreal Cognitive Assessment (MoCA) for Cognitive Screening in Old Age Psychiatry: Determining Cutoff Scores in Clinical Practice. Avoiding Spectrum Bias Caused by Healthy Controls. Int. J. Geriatr. Psychiatry.

[26] Su Y., Dong J., Sun J., Zhang Y., Ma S., Li M., Zhang A., Cheng B., Cai S., Bao Q. (2021). Cognitive Function Assessed by Mini-Mental State Examination and Risk of All-Cause Mortality: A Community-Based Prospective Cohort Study. BMC Geriatr..

[27] Patnode C.D., Perdue L.A., Rossom R.C., Rushkin M.C., Redmond N., Thomas R.G., Lin J.S. (2020). Screening for Cognitive Impairment in Older Adults: Updated Evidence Report and Systematic Review for the US Preventive Services Task Force. JAMA.

[28] Dhikav V., Jadeja B., Gupta P. (2022). Community Screening of Probable Dementia at Primary Care Center in Western India: A Pilot Project. J. Neurosci. Rural. Pract..

[29] Pashmdarfard M., Azad A. (2020). Assessment Tools to Evaluate Activities of Daily Living (ADL) and Instrumental Activities of Daily Living (IADL) in Older Adults: A Systematic Review. Med. J. Islam. Repub. Iran..

[30] Fish J., Kreutzer J.S., DeLuca J., Caplan B. (2011). Lawton-Brody Instrumental Activities of Daily Living Scale. Encyclopedia of Clinical Neuropsychology.

[31] Dias F.L.D.C., Teixeira A.L., Guimarães H.C., Barbosa M.T., Resende E.D.P.F., Beato R.G., Carmona K.C., Caramelli P. (2017). Accuracy of the 15-Item Geriatric Depression Scale (GDS-15) in a Community-Dwelling Oldest-Old Sample: The Pietà Study. Trends Psychiatry Psychother..

[32] Krishnamoorthy Y., Rajaa S., Rehman T. (2020). Diagnostic Accuracy of Various Forms of Geriatric Depression Scale for Screening of Depression among Older Adults: Systematic Review and Meta-Analysis. Arch. Gerontol. Geriatr..

[33] Stone L.E., Granier K.L., Segal D.L., Gu D., Dupre M.E. (2021). Geriatric Depression Scale. Encyclopedia of Gerontology and Population Aging.

[34] Mitchell C., Rahko P.S., Blauwet L.A., Canaday B., Finstuen J.A., Foster M.C., Horton K., Ogunyankin K.O., Palma R.A., Velazquez E.J. (2019). Guidelines for Performing a Comprehensive Transthoracic Echocardiographic Examination in Adults: Recommendations from the American Society of Echocardiography. J. Am. Soc. Echocardiogr..

[35] Hindricks G., Potpara T., Dagres N., Arbelo E., Bax J.J., Blomström-Lundqvist C., Boriani G., Castella M., Dan G.-A., Dilaveris P.E. (2021). 2020 ESC Guidelines for the Diagnosis and Management of Atrial Fibrillation Developed in Collaboration with the European Association for Cardio-Thoracic Surgery (EACTS): The Task Force for the Diagnosis and Management of Atrial Fibrillation of the European Society of Cardiology (ESC) Developed with the Special Contribution of the European Heart Rhythm Association (EHRA) of the ESC. Eur. Heart J..

[36] Esteve-Pastor M.A., Roldán V., Rivera-Caravaca J.M., Ramírez-Macías I., Lip G.Y.H., Marín F. (2019). The Use of Biomarkers in Clinical Management Guidelines: A Critical Appraisal. Thromb. Haemost..

[37] Shin S.Y., Han S.-J., Kim J.-S., Im S.I., Shim J., Ahn J., Lee E.M., Park Y.M., Kim J.H., Lip G.Y.H. (2019). Identification of Markers Associated With Development of Stroke in “Clinically Low-Risk” Atrial Fibrillation Patients. J. Am. Heart Assoc..

[38] Sebastian M.J., Khan S.K., Pappachan J.M., Jeeyavudeen M.S. (2023). Diabetes and Cognitive Function: An Evidence-Based Current Perspective. World J. Diabetes.

[39] Ab-Hamid N., Omar N., Ismail C.A.N., Long I. (2023). Diabetes and Cognitive Decline: Challenges and Future Direction. World J. Diabetes.

[40] Marseglia A., Fratiglioni L., Kalpouzos G., Wang R., Bäckman L., Xu W. (2019). Prediabetes and Diabetes Accelerate Cognitive Decline and Predict Microvascular Lesions: A Population-Based Cohort Study. Alzheimers Dement..

[41] Chaudhary S. (2023). Cognitive Impairment in Diabetes Mellitus: An Exploratory Study. Int. J. Adv. Med..

[42] Hong X., Chen X., Chu J., Shen S., Chai Q., Lou G., Chen L. (2017). Multiple Diabetic Complications, as Well as Impaired Physical and Mental Function, Are Associated with Declining Balance Function in Older Persons with Diabetes Mellitus. Clin. Interv. Aging.

[43] Kinattingal N., Mehdi S., Undela K., Wani S.U.D., Almuqbil M., Alshehri S., Shakeel F., Imam M.T., Manjula S.N. (2023). Prevalence of Cognitive Decline in Type 2 Diabetes Mellitus Patients: A Real-World Cross-Sectional Study in Mysuru, India. J. Pers. Med..

[44] Pott-Junior H., Cominetti C., Gutierrez Zuniga R., Romero-Ortuno R. (2022). Type 2 Diabetes Mellitus Predicts Cognitive Decline: Evidence from The Irish Longitudinal Study on Ageing (TILDA). Diabetes Epidemiol. Manag..

[45] Abdellatif G.A., Hassan A.M., Gabal M.S., Hemeda S.A., El-Chami N.H., Salama I.I. (2020). Mild Cognitive Impairment among Type II Diabetes Mellitus Patients Attending University Teaching Hospital. Open Access Maced. J. Med. Sci..

[46] Chow Y.Y., Verdonschot M., McEvoy C.T., Peeters G. (2022). Associations between Depression and Cognition, Mild Cognitive Impairment and Dementia in Persons with Diabetes Mellitus: A Systematic Review and Meta-Analysis. Diabetes Res. Clin. Pract..

[47] Fung A.C.H., Tse G., Cheng H.L., Lau E.S.H., Luk A., Ozaki R., So T.T.Y., Wong R.Y.M., Tsoh J., Chow E. (2018). Depressive Symptoms, Co-Morbidities, and Glycemic Control in Hong Kong Chinese Elderly Patients with Type 2 Diabetes Mellitus. Front. Endocrinol..

[48] Tudoran M., Tudoran C., Ciocarlie T., Giurgi-Oncu C. (2020). Aspects of Diastolic Dysfunction in Patients with New and Recurrent Depression. PLoS ONE.

[49] Chen L.Y., Norby F.L., Gottesman R.F., Mosley T.H., Soliman E.Z., Agarwal S.K., Loehr L.R., Folsom A.R., Coresh J., Alonso A. (2018). Association of Atrial Fibrillation With Cognitive Decline and Dementia Over 20 Years: The ARIC-NCS (Atherosclerosis Risk in Communities Neurocognitive Study). J. Am. Heart Assoc..

[50] Shanmugavaradharajan V., Balaji D., Vasu D. (2022). Cognitive Changes Associated with Hypercholesterolemia in Type 2 Diabetes Mellitus. Int. J. Med. Rev. Case Rep..

[51] Xia S.-S., Xia W.-L., Huang J.-J., Zou H.-J., Tao J., Yang Y. (2020). The Factors Contributing to Cognitive Dysfunction in Type 2 Diabetic Patients. Ann. Transl. Med..

[52] Ma L., Yuan Y.-X., Cheng F.-J., Liu Y., Wei Q., Peng Y.-F., Wang Y. (2024). The Association between Blood Lipids and Cognitive Impairment in Type 2 Diabetes Mellitus. Eur. J. Med. Res..

[53] Bulut A., Avci B. (2019). Carotid Intima-Media Thickness Values Are Significantly Higher in Patients with Prediabetes Compared to Normal Glucose Metabolism. Medicine.

[54] Roumeliotis A., Roumeliotis S., Panagoutsos S., Theodoridis M., Argyriou C., Tavridou A., Georgiadis G.S. (2019). Carotid Intima-Media Thickness Is an Independent Predictor of All-Cause Mortality and Cardiovascular Morbidity in Patients with Diabetes Mellitus Type 2 and Chronic Kidney Disease. Ren. Fail..

[55] Casey S.L., Lanting S.M., Chuter V.H. (2020). The Ankle Brachial Index in People with and without Diabetes: Intra-Tester Reliability. J. Foot Ankle Res..

[56] Mishra N. (2021). Use of ABI to Detect Peripheral Arterial Disease in Diabetes—A Recommendation for Primary Care Physicians. J. Fam. Med. Prim. Care.

[57] Militaru A., Zus S., Cimpean A.M., Iurciuc S., Matusz P., Iurciuc M., Lighezan D., Militaru M. (2019). Early Diagnosis of Cardiotoxicity in Patients Undergoing Chemotherapy for Acute Lymphoblastic Leukemia. Anticancer. Res..

[58] Liu L., Hayden K.M., May N.S., Haring B., Liu Z., Henderson V.W., Chen J.-C., Gracely E.J., Wassertheil-Smoller S., Rapp S.R. (2022). Association between Blood Pressure Levels and Cognitive Impairment in Older Women: A Prospective Analysis of the Women’s Health Initiative Memory Study. Lancet Healthy Longev..

[59] Wood K.A., Han F., Ko Y.-A., Wharton W. (2022). Do Sex Differences Exist in Cognitive Function in Patients with Atrial Fibrillation?. Alzheimer’s Dement..

[60] Militaru M., Rachieru C., Lighezan D.F., Militaru A.G. (2021). The Impact of Hypertension and Atrial Fibrillation on Cognitive Decline and Subclinical Atherosclerosis. Brain Sci..

[61] Proietti M., Farcomeni A. (2018). Association Between Peripheral Artery Disease and Incident Risk of Atrial Fibrillation: Strong Evidence Coming from Population-Based Cohort Studies. J. Am. Heart Assoc..

[62] Lu Y.-C., Wang C.-P., Hung W.-C., Wu C.-C., Yu T.-H., Hsu C.-C., Wei C.-T., Chung F.-M., Lee Y.-J., Tang W.-H. (2022). Interactive Effect of Obesity and Cognitive Function Decline on the Risk of Chronic Kidney Disease Progression in Patients with Type 2 Diabetes Mellitus: A 9.1-Year Cohort Study. Int. J. Med. Sci..

[63] Ghoshal S., Allred N.D., Freedman B.I. (2020). The Contribution of Kidney Disease to Cognitive Impairment in Patients with Type 2 Diabetes. Curr. Diab Rep..

